# Electrochemical HPLC Determination of Piperazine Antihistamine Drugs Employing a Spark-Generated Nickel Oxide Nanoparticle-Modified Carbon Fiber Microelectrode

**DOI:** 10.1021/acsomega.3c09474

**Published:** 2024-01-20

**Authors:** Zeynab Belbasi, Jan Petr, Juraj Sevcik, David Jirovsky, Jan Hrbac

**Affiliations:** † Faculty of Science, Department of Analytical Chemistry, Palacky University, 17. Iistopadu 12, 771 46 Olomouc, Czech Republic; ‡ Faculty of Science, Department of Chemistry, Masaryk University, Kamenice 5, 625 00 Brno, Czech Republic

## Abstract

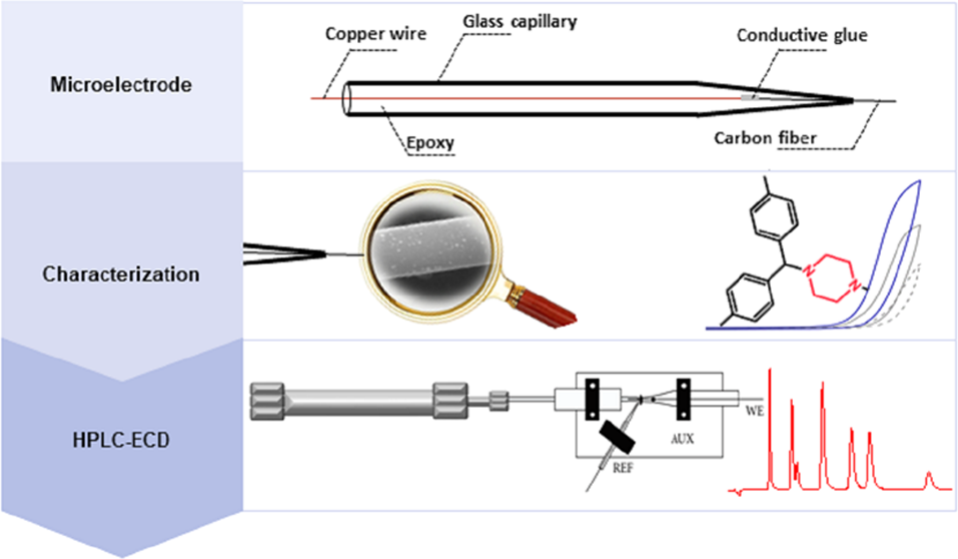

In this work, we demonstrate a sensitive high-performance liquid chromatography (HPLC) method for the determination of piperazine antihistamine drugs employing innovative electrochemical detection based on a spark-generated nickel oxide nanoparticle-modified carbon fiber microelectrode built into a miniaturized electrochemical detector. The direct carbon fiber-to-nickel plate electrode spark discharge, carried at 0.8 kV DC, with the nickel electrode connected to the negative pole of the high-voltage power supply, provides extremely fast (1 s) in situ tailoring of the carbon fiber microelectrode surface by nickel oxide nanoparticles. It has been found that nickel oxide nanoparticles exhibit an electrocatalytic effect toward the piperazine moiety electrooxidation process, as confirmed by voltammetric experiments, revealing the shift in the peak potential from 1.25 to 1.09 V versus Ag/AgCl. Cetirizine, cyclizine, chlorcyclizine, flunarizine, meclizine, and buclizine were selected as sample piperazine antihistamine drugs, while diclofenac served as an internal standard. The isocratic reversed-phase separation of the above set was achieved within 15 min using an ARION-CN 3 μm column with a binary mobile phase consisting of 50 mM phosphate buffer (pH 3) and methanol (45/55, v/v). The limits of detection (LOD) were within the range of 3.8–120 nM (for cyclizine and buclizine) at *E* = +1500 mV (vs Ag/AgCl), while the response was linear within the concentration range measured up to 5 μmol L^–1^. The method was successfully applied to the determination of piperazine antihistamine drugs in spiked plasma samples.

## Introduction

Histamine as a primary endogenous ligand of H1–H4 G protein-coupled receptors plays a significant role in the stimulation of smooth muscle contraction, gastric acid secretion, neurotransmission, hemopoiesis, and cell proliferation. However, its most notable effects include immune modulation and allergic inflammation.^1^ Antihistamine drugs are histamine antagonists and most commonly target the H1 receptor,^2^ providing antiallergic and anti-inflammatory effects that can be utilized in the treatment of a variety of allergies and vestibular disorders, but they can also act as sedatives, sleeping aids, and antiemetics.^3^

**Figure 1:**
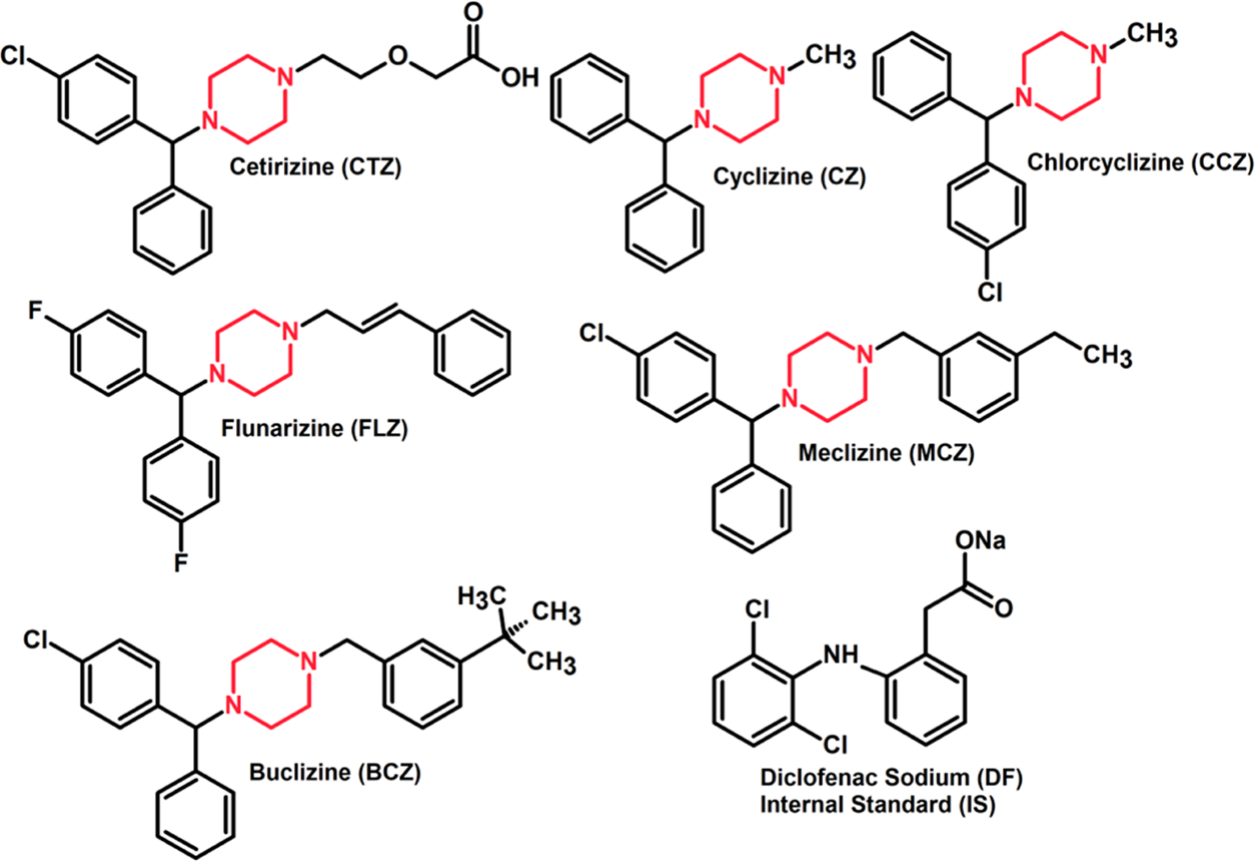
Chemical structures of the studied antihistamine drugs and of the diclofenac internal standard.

Cyclizine, chlorcyclizine, cetirizine, flunarizine, meclizine, and buclizine belong to H1-antihistamine piperazine derivatives (for structures, see Figure 1). All these drugs control blood pressure and have effects on the central nervous system.^4^ Moreover, cetirizine is effective in the treatment of allergic rhinitis, pollen hypersensitivity, sneezing, and itching.^5^ Motion sickness can be prevented by using chlorcyclizine, meclizine, and buclizine.^6,7^ Cyclizine is used for the management of nausea and vomiting,^8^ while flunarizine is administered for the treatment of vertigo of central or peripheral origin.^9,10^

Piperazine antihistamines may exhibit side effects, including sedation, weakness, depression, drowsiness, decreased cognitive processing, cardiac failure, and tachycardia. Overdose can have a detrimental impact on breathing and can cause swelling of the throat or tongue.^5,11,12^ Hence, the determination of antihistamine drugs in blood is of immense importance. To this end, high-performance liquid chromatography (HPLC) is frequently used, most often in combination with spectrophotometric detectors^13−16^ or coupled to mass spectrometry (HPLC–MS).^17,18^ In this work, we investigate the possibility of applying a nanomaterial-based electrochemical detector (HPLC-ECD) offering enhanced sensitivity compared to both MS and spectrophotometry.

Electrochemical detection represents a cost-effective solution in the analysis of electroactive compounds such as various biomarkers,^19^ neurotransmitters,^20,21^ nucleic acids,^22^ antioxidants,^23^ saccharides,^24^ drugs,^25^ etc. As ECD probes only species that are electroactive at the applied potential, it often offers higher selectivity than spectrophotometric detection^26−28^ and much lower running costs compared with MS.^11,29^ Nevertheless, the determinations of low concentrations of analytes that require the use of high detection potentials are particularly challenging. Piperazine antihistamines fall into this category, as they are oxidizable at relatively high potentials, approaching the anodic potential limit of common electrode materials. The most widely studied of these drugs is cetirizine, which exhibits a pH-dependent irreversible cyclic voltammetry (CV) peak with a slope of *E*_p_ versus pH at around 59 mV/pH, suggesting that an equivalent number of protons and electrons participate in the electrochemical transformation. So far, the determination of cetirizine using various modified electrodes based on carbon nanotubes (CNTs),^30^ AgNP/TiO_2_/CNTs,^31^ PtNP/CNTs,^32^ poly(l-leucine)/CNTs,^33^ ZnO/graphene,^34^ or NiCo NPs^35^ has been proposed. In all these electrodes, the cetirizine peak position shifts from ca. 1.1 V versus Ag/AgCl at pH = 3 down to approximately 0.8 V at pH = 8. The electrooxidation mechanism suggested in the literature features the formation of a primary N-centered radical followed by deprotonation of the neighboring α-carbon and the transfer of the unpaired electron to the neighboring α-carbon.^6,31,32,36^ The final step is the dimerization of the two C-centered radicals (Figure S1).

The electrochemistry of other drugs in the set has been far less studied. Meclizine was determined on screen-printed electrodes (SPEs) in plain or Fe_2_O_3_ nanocubes modified with or without sodium dodecyl sulfate as an additive.^11^ In 0.05 M H_2_SO_4_, it provided an irreversible peak at 1.5 V versus Ag pseudoreference electrode. The voltammetric behavior of flunarizine in 0.5 M H_2_SO_4_ with a 20% methanol mixture was also studied on a glassy carbon electrode^37^ and found to give an irreversible peak at 1.3 V versus SCE. There is no literature dealing with the voltammetry of cyclizine and chlorcyclizine. However, an HPLC method with coulometric detection was proposed for cyclizine and its demethylated metabolite norcyclizine, with chlorcyclizine being used as an internal standard.^38^ The hydrodynamic voltammogram of cyclizine can be found in ref, (39) where cyclizine was used as an internal standard in the determination of captopril. The onset potential was 0.6 V, and the plateau was reached at ca. 0.9 V versus hydrogen–palladium reference electrode (i.e., approximately 0.9 and 1.2 V vs Ag/AgCl).

In this work, we aim to pioneer a novel approach in developing a simple, sensitive, and isocratic HPLC method for the simultaneous determination of five antihistamine piperazine drugs in blood, based on a miniaturized electrochemical detector featuring a carbon fiber microelectrode directly inserted in the HPLC column output capillary. The novelty lies in the modification of the carbon fiber microelectrode achieved through the direct spark discharge method, specifically the carbon fiber-to-carbon plate electrode or carbon fiber-to-nickel plate electrode technique at 0.8 kV under ambient conditions. Based on scanning electron microscopy/energy-dispersive X-ray (SEM-EDX) data, the latter case resulted in the in situ tailoring of the carbon fiber microelectrode surface with nickel oxide nanoparticles under an extremely fast (the sparking process takes 1 s), green (no liquids or organic solvents are used), and cost-effective (no reducing compounds, stabilizers, or templates are used) approach. Voltammetry demonstrated that nickel oxide nanoparticles exhibit a remarkable electrocatalytic effect toward the piperazine moiety electrooxidation.

## Experimental Section

### Materials

The standards of antihistamines (AHs) including buclizine dihydrochloride (BCZ; 1-[(4-*tert*-butylphenyl)methyl]-4-[(4-chlorophenyl)-phenylmethyl]piperazine dihydrochloride), purity ≥98%; meclizine dihydrochloride (MCZ; 1-[(4-chlorophenyl)-phenylmethyl]-4-[(3-methylphenyl)methyl]piperazine dihydrochloride), ≥97%; cetirizine dihydrochloride (CTZ; [2-[4-[(4-chlorophenyl)phenylmethyl]-1-piperazinyl]ethoxy]acetic acid dihydrochloride), ≥98%; flunarizine dihydrochloride (FLZ; 1-[bis(4-fluorophenyl)methyl]-4-[(*E*)-3-phenylprop-2-enyl]piperazine dihydrochloride), ≥98%; cyclizine hydrochloride (CZ; 1-benzhydryl-4-methylpiperazine dihydrochloride), 97.6%; chlorocyclizine (CCZ; 1-[(4-chlorophenyl)-phenylmethyl]-4-methylpiperazine dihydrochloride), ≥98%; diclofenac sodium salt (DF; sodium 2-[2-(2,6-dichloroanilino)phenyl]acetate, ≥98%, used as an internal standard—IS), and bovine calf serum (BCS) were purchased from Sigma. Stock solutions of analytes (5 mg/mL) were prepared by dissolution in methanol and water 50/50 (v/v). For mobile phase preparation, sodium dihydrogen phosphate (TraceSelect purity, Fluka), potassium monohydrogen phosphate (Sigma), methanol, and acetonitrile (HPLC-grade, Merck) were used.

### Fabrication and Modification of Carbon Fiber Microelectrodes (CFMEs)

The carbon fiber microelectrodes were fabricated using individual carbon fibers (7 μm in diameter, Kordcarbon a.s., Czech Republic) following the method described in ref^40^. The length of the active part of carbon fiber was ca. 5–6 mm. For electrode modification, spark discharge was conducted between the CFME and the graphite or nickel wire electrode at 0.8 kV, with the CFME connected to the positive pole of an in-house-built DC high-voltage power supply.^41−43^ Electrical discharges were performed at ambient conditions unless specified otherwise; the sparking resulted in trimming the length of the fiber to ca. 4–5 mm.

### Scanning Electron Microscopy (SEM) and Energy-Dispersive X-ray Analysis (EDX)

The morphology of carbon fibers was inspected by using a Vega3 microscope (Tescan, Czech Republic). CFs were cut off from the CFMEs and placed on an aluminum holder covered by conductive carbon adhesive tape. The elemental composition of CFs was determined by using the Quantax EasyEDS module (Bruker).

### Electrochemical Measurements

Electrochemical experiments (cyclic voltammetry and amperometry) were performed on a Nanoampere electrochemical workstation (L-Chem, Czech Republic) in a three-electrode system in a single-compartment cell. A leak-free Ag/AgCl electrode (LF-2, Innovative Instruments, Inc.) served as a reference electrode, a platinum wire as an auxiliary electrode, and CFMEs as working electrodes. The electrocatalytic properties of sparked nickel oxide nanoparticles were investigated by cyclic voltammetry using graphite SPEs, which were in-house fabricated on a 175 μm thick polyester substrate using Loctite EDAG PF 407 A ink (details are given in ref (42)). Amperometric experiments were performed in stirred solutions (using a magnetic stirrer at 200 r.p.m.). The analyte (cetirizine as a candidate of the selected AHs) was injected manually with a 20 μL Hamilton syringe. The measurements were conducted in 50 mM NaH_2_PO_4_ (pH 3)/MeOH (45/55, v/v) at room temperature.

### Chromatographic System

The HPLC system consisted of an ESA isocratic pump (Model 582, ESA Inc., Chelmsford, MA) and a Rheodyne manual injector equipped with a 20 μL loop. A Coulochem III potentiostat (ESA Inc., Chelmsford, MA) operating in a three-electrode arrangement was coupled to a self-designed amperometric flow cell^43^ equipped with a CFME. Prior to the injector, an ESA guard cell (Model 5020) was placed. The samples were introduced into the system using glass injection syringes (Hamilton, Reno, NV). HPLC separations were performed using a reversed-phase ARION-CN 3 μm column, 150 mm × 2.1 mm I.D. The final mobile phase consisting of 50 mM NaH_2_PO_4_ (pH 3)/MeOH (45/55, v/v) was filtered and degassed by helium sparging before use. The flow rate was 0.15 mL min^–1^. The chromatograms were recorded and processed using a Clarity Chromatographic Station (DataApex, Prague, Czech Republic).

### Sample Preparation

A simple procedure was used for the processing of plasma samples, as follows: 100 μL of bovine calf serum (BCS) spiked with analytes and diclofenac (IS) were mixed with 100 μL of acetonitrile for protein removal. The mixture was vortexed, sonicated, and centrifuged (at 7200*g*; 5 min) to separate the precipitated proteins. The supernatant was transferred into a new vial, diluted (50/50, v/v) with 25 mM phosphate buffer (pH 3), and injected into the HPLC system.

## Results and Discussion

### Modification of CFME by Spark Discharge

**Figure 2:**
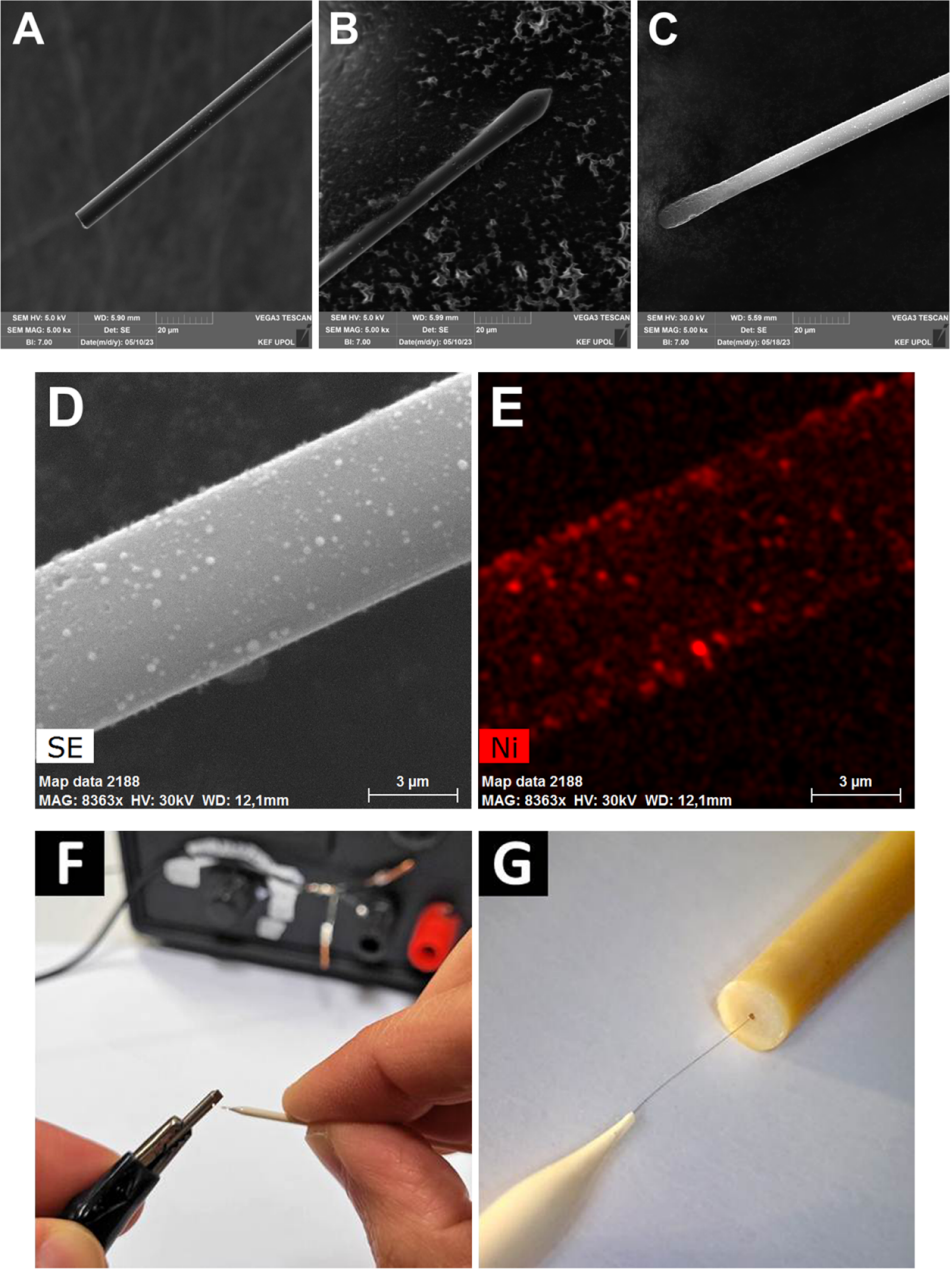
SEM images of bare carbon fibers (A), carbon-sparked carbon fibers (B), and nickel-sparked carbon fibers (C). Section of Ni-sparked carbon fiber (D) used for EDX mapping of nickel (E). Spark discharge modification procedure (F) and spark-modified CFME installed in the HPLC capillary (0.005’’ I.D.) outlet (G).

The surface of the carbon fiber microelectrodes must be activated before use. The principle of activation is the removal of the eventual thin polymer coating on the CFME surface, produced during the manufacture (“sizing”), and the introduction of oxygen-containing moieties (–OH, –COOH, –C=O, –COC–, etc.), resulting in defined and reproducible electrochemical behavior. For activation, electrochemical pretreatment is predominantly used, although in the literature on CFMEs, a microburner flame,^44^ laser irradiation,^45^ or spark discharge between CFME and the tungsten electrode^46^ is also used for simultaneous activation and machining the end of the CFME into the shape of a conical tip. A microspark discharge between CFME and lower-melting-point metals appears to be an advantageous approach, as the CFME surface can be simultaneously activated by the heat produced during the electrical discharge and at the same time modified with spark-generated nanomaterials derived from the material of (eroded) counter-electrode (i.e., a source electrode, see also ref (16)). In this work, we sparked CFMEs with Pt, Au, Ag, Cu, Ni, and C and assessed their amperometric responses to the electrooxidation of cetirizine, which was selected as a candidate for the studied antihistamine drugs. The best sensing properties were found for Ni-sparked CFMEs, which were studied further. Figure 2 shows the SEM images of bare (panel A), carbon-sparked (B), and Ni-sparked CFMEs (C). The presence of Ni oxide nanoparticles (NPs) attached to carbon fiber was confirmed by the EDX spectrum, while the coverage by nickel was visualized by the EDX map (E). The illustration of spark discharge modification of carbon fiber, done by manually bringing the tip of the CFME into close vicinity to the source electrode so that the discharge occurs, is shown in Figure 2F. This modification procedure is simple and fast (<1 s), efficient, and waste-free, as it does not require any chemicals, solvents, etc., necessary for conventional synthesis of nickel/nickel oxide nanoparticles.

### Electrocatalysis of Cetirizine by Spark Discharge Generated Nickel Oxide Nanoparticles

Since the preliminary screening of the various (metal or carbon) sparked CFMEs gave prominence to Ni-sparked CFMEs, their electrocatalytic properties with respect to C-sparked CFMEs, which was used for comparison, were further studied by cyclic voltammetry and cetirizine as a representative model compound of the group.

**Figure 3:**
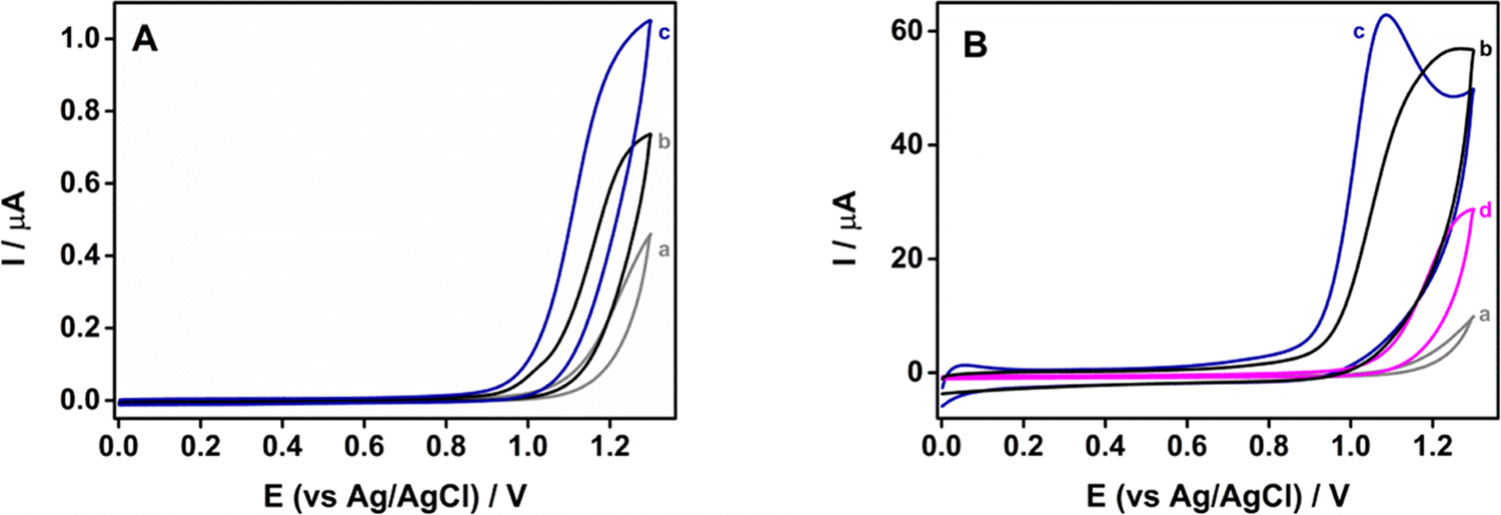
Cyclic voltammograms of 1 mM cetirizine in 0.05 M dihydrogen phosphate (pH = 3), recorded on (A) CFMEs and (B) SPEs, a: plain, b: C-sparked, and c: Ni-sparked under ambient conditions. Scan d: Ni-sparked SPE was under an argon atmosphere. Scan rate: 100 mV/s.

Figure 3A shows the representative CVs of cetirizine on bare, C-sparked, and Ni-sparked CFMEs under ambient conditions, documenting the increased sensitivity of the Ni-sparked electrode. As the modification of the CFME by NiNPs is limited to a certain area around the tip and the microelectrode, the properties of the CFME inevitably bring a significant contribution of the radial diffusion mass transport of the analyte to the electrode surface, leading to sigmoidal wave-shaped CVs rather than clearly defined peaks. Indeed, the analogous experiment was also conducted using SPEs, for which more effective modification and mass transfer by semi-infinite linear diffusion can be achieved.^43^ As shown in Figure 3, compared with C-sparked SPE (scan b), the Ni-sparked SPE provided a well-defined, higher, and sharper cetirizine oxidation CV peak, shifted toward less positive potentials (scan c). This finding clearly demonstrates the electrocatalytic effect.

Interestingly, the SPEs sparked by Ni under an inert atmosphere, where nickel and/or nickel carbide NPs are expected to be formed, provided a much smaller enhancement of the cetirizine CV response with no shift in peak potential (trace d). From this result, it follows that the likely electrocatalyst is nickel oxide nanoparticles. In the previous study, the composition of the Ni component of ambient air Ni-sparked SPE was shown to contain 50% Ni_2_O_3_, 18% NiO, and 32% Ni(OH)_2_, as determined by X-ray photoelectron spectroscopy (XPS).^42^ The detailed investigation of the electrocatalytic effect (electrode kinetics and mechanism, pH dependence, etc.) will be the subject of future studies.

### Chromatographic Analysis

**Figure 4:**
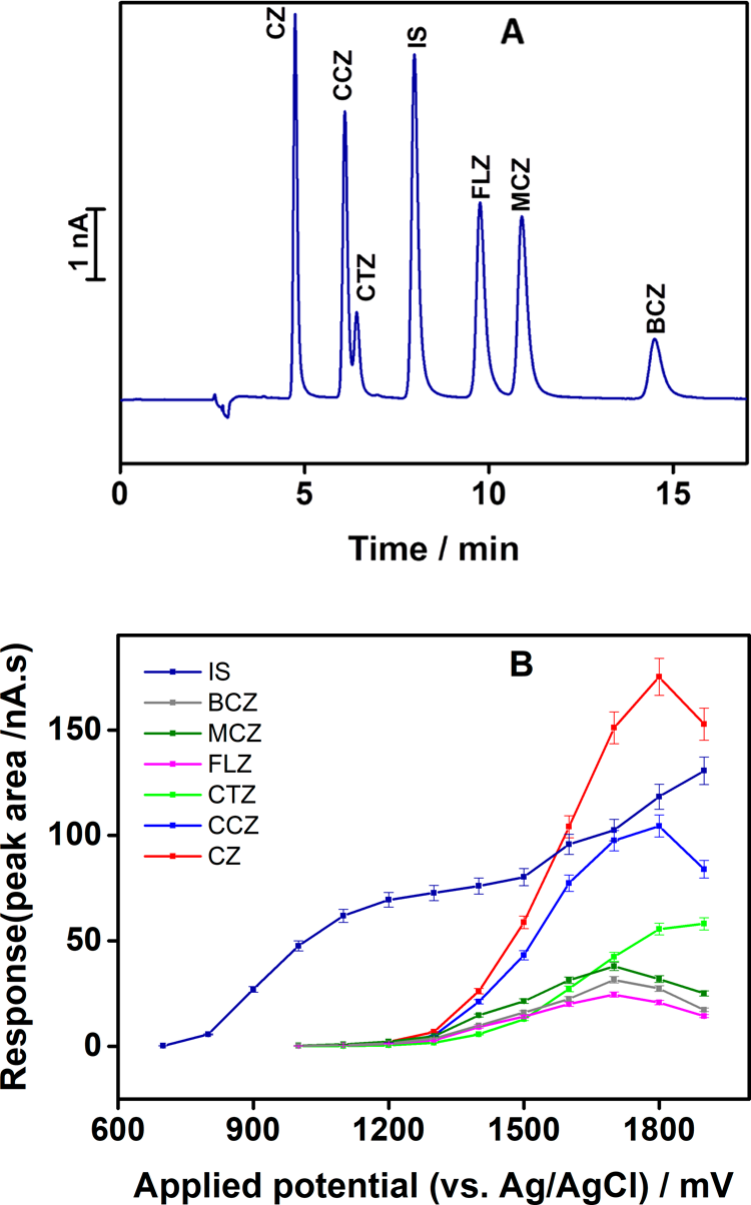
(A) Chromatogram of analytes with an internal standard under selected experimental conditions (mobile phase 50 mM NaH_2_PO_4_ (pH 3)/MeOH (45/55, v/v); *E*_applied_ = 1500 mV, *c*_AHs,IS_ = 5 × 10^–6^ mol L^–1^) and (B) corresponding hydrodynamic voltammograms of all studied drugs and IS, *c* = 7 μM each).

HPLC separations employing electrochemical detection are usually restricted by the use of isocratic elution exclusively, where there are no undesired changes in the conductivity of the eluent over time, typically producing considerable drift of the baseline. However, this often poses a significant limitation in the simultaneous analysis of substances of different polarities. After testing several HPLC columns packed with commonly used sorbents (C18, C8), an ARION-CN 3 μm (150 mm × 2.1 mm I.D.) column was used for the purpose of this study, as it provided the most compact separation for the set of antihistamines, including the favorable central position of the IS. As a part of the optimization process, the effects of column temperature, buffer pH and its concentration, the organic modifier type, and its content on the chromatographic behavior of the examined compounds were also investigated. A mobile phase containing 50 mM phosphate buffer at pH 3 provided both good retention and good selectivity for the selected analytes. Because of the higher peak resolution obtained, methanol was preferred as an organic modifier over acetonitrile. The final mobile phase consisting of 50 mM NaH_2_PO_4_ (pH 3)/MeOH (45/55, v/v) was used for HPLC experiments. Except for the pair of CCZ and CTZ, a baseline separation was achieved for the studied piperazine antihistamines, including IS, within 15 min (Figure 4(A)). However, it would be possible to achieve complete resolution of these two substances as well, but at the cost of prolonged analysis time.

### Hydrodynamic (HD) Voltammetry

To define the optimum working potential for the compounds of interest, we conducted hydrodynamic voltammetry measurements under the selected chromatographic conditions. HD voltammograms (HDVs) were obtained by repeatedly injecting a standard mixture (7 μM each) into an HPLC system at gradually increased working potential (Figure 4(B)). As also evident from the recorded HDVs, the chosen antihistamines are electrochemically oxidizable at high positive potentials. As can be expected, they exhibit similar HDV profiles except for diclofenac (IS), which has a different chemical structure, and much lower potentials are needed for its oxidation. A working potential of +1500 mV (vs Ag/AgCl) provided the optimum analytical response for the simultaneous determination of the examined piperazine antihistamine drugs and the internal standard.

The onset potentials for the studied piperazine antihistamine oxidation responses (ca. 1250 mV vs Ag/AgCl) are higher than those found from the voltammetric measurements (900 mV). This can be attributed to an IR drop in the flow cell.

### Analytical Figures of Merit and Analytical Utility

Calibration curves assessed as peak area ratios of corresponding AHs/IS versus concentration AHs were plotted, and basic parameters for the partial validation of the method were calculated (Table 1). LOD and LOQ values for the studied compounds measured under the selected experimental conditions were obtained by using the signal-to-noise method. Values of *S*/*N* = 3 and 10 were used to determine the LOD and LOQ, respectively. Although microelectrodes have not been cleaned or regenerated between analyses, the results indicate good linearity of this method for both intra- and interday assays.

**Table 1:** Results of the Partial Validation of the Method

analyte	intraday^a^	interday^b^	reproducibility (different electrodes^b^)	LOD (M)	LOQ (M)	slope^c^	intercept	*R*^2^
	area RSD (%)	area RSD (%)	area RSD (%)					
CZ	3.9	2.5	6.9	3.8 × 10^–9^	1.3 × 10^–8^	0.12946	0.00625	0.99964
CCZ	4.7	4.9	10.3	6.0 × 10^–9^	2.0 × 10^–8^	0.09986	0.00661	0.99942
CTZ	6.8	21.5	25.5	2.8 × 10^–8^	9.2 × 10^–8^	0.02196	0.000037	0.99879
FLZ	3.7	5.9	10.5	3.2 × 10^–8^	1.1 × 10^–7^	0.15173	0.04517	0.99083
MCZ	3.2	7.3	11.3	2.4 × 10^–8^	8.0 × 10^–8^	0.15073	0.02401	0.99715
BCZ	11.3	38.8	14.0	1.2 × 10^–7^	4.0 × 10^–7^	0.07162	0.04099	0.96472

a *n* = 5, concentration = 5 × 10^–6^ M.

b *n* = 4, concentration = 5 × 10^–6^ M.

c The upper concentration limit of the calibration curves was 5 × 10^–6^ M for all studied compounds.

The interelectrode reproducibility has been evaluated using four individual Ni-sparked CFMEs (Table 1). It should be noted that the length of the CFME inserted into the HPLC capillary outlet has a significant impact on overall analytical performance. Since this parameter is very difficult to control during the manual assembly of the flow cell and, hence, the same active length cannot be maintained for each of the electrodes tested, the reported data could be burdened with this error. While we likely could have expected better reproducibility when comparing individual electrodes using amperometry at batch conditions (i.e., without placing them in an HPLC flow cell), we found the results satisfactory.

**Figure 5:**
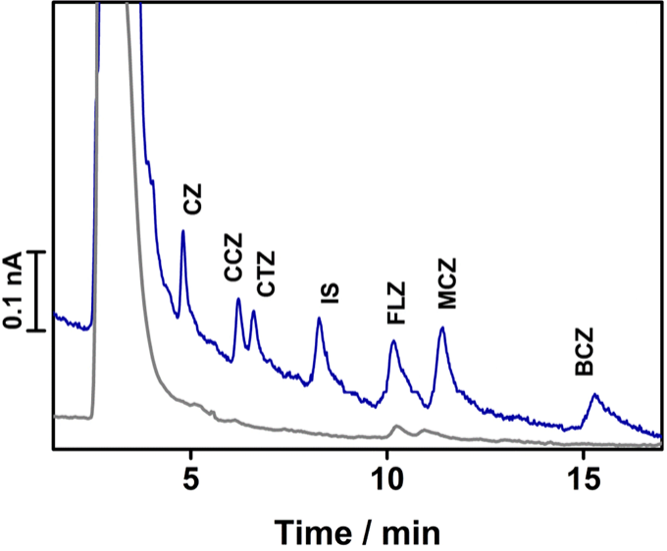
Chromatogram of plasma spiked with analytes and an internal standard (*c*_CZ,CCZ,IS_ = 2 × 10^–7^ mol L^–1^, *c*_CTZ_ = 5 × 10^–7^ mol L^–1^, *c*_FLZ,MCZ_ = 8 × 10^–7^ mol L^–1^, *c*_BCZ_ = 2 × 10^–6^ mol L^–1^) shown along with the blank plasma (gray trace). For chromatographic conditions, see the legend of Figure 4.

To our knowledge, not many electrochemistry-based methods can be found in the literature, especially when combined with the separation method, which allows a simultaneous analysis of multiple antihistamines. Some of the currently published analytical techniques and the LOD achieved are summarized in Table S1. To test the applicability of the method, the determination of antihistamine drugs in spiked bovine calf serum samples was carried out. Figure 5 shows the complete chromatographic separation of all of the analytes in bovine calf serum.

## Conclusions

The research presented an innovative HPLC method designed for the simultaneous determination of piperazine antihistamine drugs employing amperometric detection via nickel spark-modified CFMEs. This methodology uncovered a notable electrocatalytic effect of spark-generated Ni oxide nanomaterials on the electrooxidation process of the piperazine moiety as evaluated through voltammetric experiments. The proposed method revealed advantages, including simplicity, low cost, and high sensitivity for detection of antihistamines, compounds with high oxidation potentials. The detection limits after chromatographic separation, determined at *E* = +1500 mV (vs Ag/AgCl), were 3.8 nmol L^–1^ for cyclizine, 28 nmol L^–1^ for cetirizine, 6.0 nmol L^–1^ for chlorcyclizine, 32 nmol L^–1^ for flunarizine, 24 nmol L^–1^ for meclizine, and 120 nmol L^–1^ for buclizine. The response was linear within the concentration range measured up to 5 μmol L^–1^. The developed HPLC-ECD method was successfully applied to spiked plasma samples. The benefit of the methodology described above is its greenness due to the downsizing of key procedures. Toxic chemicals were avoided whenever possible during the workflow. CFME modification by nickel oxide nanoparticles was done using the spark discharge procedure, which is fast and requires only high voltage and nickel metal electrodes. In HPLC separation, the consumption of organic solvents was significantly reduced.

## Supporting Information Available

Mechanism of CTZ electrooxidation; and literature overview of analytical techniques for the studied antihistamines (PDF)
