# Supplementary material for: Electrochemical HPLC Determination of Piperazine Antihistamine Drugs Employing a Spark-Generated Nickel Oxide Nanoparticle-Modified Carbon Fiber Microelectrode
Source: ACS Omega. 2024 Jan 20;9(4):5038–45. doi: 10.1021/acsomega.3c09474 (PMC10831984; doi:10.1021/acsomega.3c09474)
Supplement: Supplementary file 1 — ao3c09474_si_001.pdf [file ao3c09474_si_001.pdf]

Supplementary Data

Electrochemical HPLC determination of piperazine antihistamine drugs employing spark-generated nickel oxide nanoparticle-modified carbon fiber microelectrode

Zeynab Belbasi[a], Jan Petr[a], Juraj Sevcik[a], David Jirovsky[a,*], Jan Hrbac[a,b*]

[a]*Palacky University, Faculty of Science, Department of Analytical Chemistry, 17. listopadu 12, 771 46 Olomouc, Czech Republic*
[b]*Masaryk University, Faculty of Science, Department of Chemistry, Kamenice 5, 625 00 Brno, Czech Republic*
*Corresponding author. E-mail: david.jirovsky@upol.cz (D.J.), jhrbac@atlas.cz (J.H.)

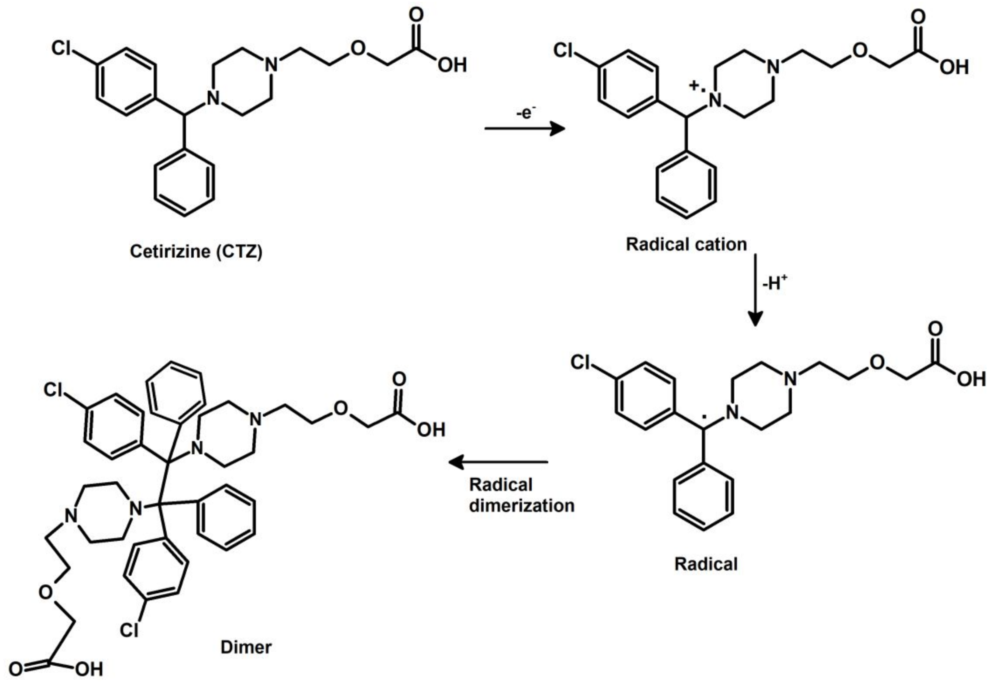

Fig S1. Mechanism of CTZ electrooxidation [1]

Table S1

Overview of published analytical techniques and LODs reached

Analyte	Technique	LOD	Reference
CZ	LC–MS/MS	3.8×10^{-9} M	[2]
	Spectrofluorimetric	1.1×10^{-8} M	[3]
	Potentiometric determination by a PVC membrane sensor	4.5×10^{-6} M	[4]
CZZ	MLC- UV	1.1×10^{-6} M	[5]
	HPLC- UV	5.9×10^{-7} M	[6]
CTZ	Square wave voltammetry (SWV) at disposable pretreated graphite pencil electrode	1.6×10^{-7} M	[7]
	Voltammetric analysis at poly(L-leucine) layered carbon nanotube paste electrode	1.7×10^{-7} M	[8]
	Cyclic voltammetry (CV) at Poly (DL-valine) electro-polymerized carbon nanotube paste (PVLMCNTPS)	1.1×10^{-7} M	[9]
	CV at glassy carbon electrode modified with multi-walled carbon nanotubes	7.1×10^{-8} M	[10]
	Adsorptive stripping DPV at multiwalled carbon nanotube-platinum nanoparticles nanocomposite modified carbon paste electrode	5.9×10^{-8} M	[1]
	Square-wave adsorptive anodic stripping voltammetry (SWAdAS) at carbon black-modified electrode	4.0×10^{-7} M	[11]
	Differential pulse voltammetry (DPV) at glassy carbon electrode	4.5×10^{-6} M	[12]
	HPLC- UV	6.5×10^{-8} M	[13]
	HPLC- UV	2.1×10^{-7} M	[14]
	HPLC- UV	2.1×10^{-7} M	[15]
FLZ	SWV at modified glassy carbon electrode (TiO_2/IL/GCE)	7.4×10^{-8} M	[16]
	HPLC- UV	2.2×10^{-7} M	[17]
	MLC- UV	4.9×10^{-7} M	[5]
MCZ	DPV at disposable screen-printed electrodes modified with uniform iron oxide nanocubes	1.7×10^{-6} M	[18]
	CV at unmodified/bare graphite screen-printed electrodes (GSPE)	8.0×10^{-8} M	[19]
	HPLC- UV	4.0×10^{-6} M	[20]
BCZ	HPLC- UV	1.2×10^{-7} M	[21]
	HPLC- UV	1.9×10^{-7} M	[22]

References

1. Kalambate, P.K. and A.K. Srivastava, *Simultaneous voltammetric determination of paracetamol, cetirizine and phenylephrine using a multiwalled carbon nanotube-platinum nanoparticles nanocomposite modified carbon paste electrode.* Sensors and Actuators B: Chemical, 2016. **233**: p. 237-248.
2. Mohammadi, A., et al., *An LC-MS-MS method for the determination of cyclizine in human serum.* J Chromatogr B Analyt Technol Biomed Life Sci, 2005. **824**(1-2): p. 148-52.
3. Fares, M.Y., et al., *Spectrofluorimetric Approach for Quantification of Cyclizine in the Presence of its Toxic Impurities in Human Plasma; in silico Study and ADMET Calculations.* J Fluoresc, 2022. **32**(3): p. 993-1003.
4. Ganjali, M., et al., *Potentiometric Determination of Cyclizine by a PVC membrane Sensor.* INTERNATIONAL JOURNAL OF ELECTROCHEMICAL SCIENCE, 2013. **8**(8): p. 10487-10497.
5. Martinez-Algaba, C., et al., *Analysis of pharmaceutical preparations containing antihistamine drugs by micellar liquid chromatography.* J Pharm Biomed Anal, 2006. **40**(2): p. 312-21.
6. Moneghini, M., et al., *Analysis of chlorcyclizine and related compounds by liquid chromatography for stability studies.* Internarionul Journal of Pharmuceurics, 1990. **63**.
7. Karakaya, S. and D.G. Dilgin, *Low-cost determination of cetirizine by square wave voltammetry in a disposable electrode.* Monatshefte für Chemie - Chemical Monthly, 2019. **150**(6): p. 1003-1010.
8. Pushpanjali, P.A., et al., *Voltammetric analysis of antihistamine drug cetirizine and paracetamol at poly(L-Leucine) layered carbon nanotube paste electrode.* Surfaces and Interfaces, 2021. **24**: p. 101154.

9. Girish, T., et al., *Poly (DL-valine) electro-polymerized carbon nanotube paste sensor for determination of antihistamine drug cetirizine.* Journal of Electrochemical Science and Engineering, 2020.
10. Patil, R.H., R.N. Hegde, and S.T. Nandibewoor, *Electro-oxidation and determination of antihistamine drug, cetirizine dihydrochloride at glassy carbon electrode modified with multi-walled carbon nanotubes.* Colloids Surf B Biointerfaces, 2011. **83**(1): p. 133-8.
11. Lourencao, B.C., et al., *Sensitive voltammetric determination of hydroxyzine and its main metabolite cetirizine and identification of oxidation products by nuclear magnetic resonance spectroscopy.* Journal of Electroanalytical Chemistry, 2017. **807**: p. 187-195.
12. Gungor, S.D., *Electrooxidation of cetirizine dihydrochloride with a glassy carbon electrode.* Die Pharmazie - An International Journal of Pharmaceutical Sciences, 2004. **59**(12): p. 929-933.
13. Jebali, S., S. Bahri, and L. Latrous, *Estimation of measurement uncertainty and validation of RP-HPLC for simultaneous determination of five antihistamines in pharmaceutical formulations.* Accreditation and Quality Assurance, 2020. **25**(2): p. 147-159.
14. Karakus, S., I. Kucukguzel, and S.G. Kucukguzel, *Development and validation of a rapid RP-HPLC method for the determination of cetirizine or fexofenadine with pseudoephedrine in binary pharmaceutical dosage forms.* J Pharm Biomed Anal, 2008. **46**(2): p. 295-302.
15. Jaber, A.M., et al., *Determination of cetirizine dihydrochloride, related impurities and preservatives in oral solution and tablet dosage forms using HPLC.* J Pharm Biomed Anal, 2004. **36**(2): p. 341-50.
16. Singh, K., N. Jadon, and R. Jain, *Synergistic effect of 1-butyl-2,3-dimethylimidazolium bis (trifluoromethanesulfonyl) imide and titanium oxide on the redox behaviour of flunarizine in solubilized media.* Colloids Surf B Biointerfaces, 2018. **166**: p. 72-78.
17. Busaranon, K., W. Suntornsuk, and L. Suntornsuk, *Comparison of UV spectrophotometric method and high performance liquid chromatography for the analysis of flunarizine and its application for the dissolution test.* J Pharm Biomed Anal, 2006. **41**(1): p. 158-64.
18. Khorshed, A.A., et al., *Disposable screen-printed electrodes modified with uniform iron oxide nanocubes for the simple electrochemical determination of meclizine, an antihistamine drug.* Analytical Methods, 2019. **11**: p. 282-287.
19. Khorshed, A.A., M. Khairy, and C.E. Banks, *Voltammetric determination of meclizine antihistamine drug utilizing graphite screen-printed electrodes in physiological medium.* Journal of Electroanalytical Chemistry, 2018. **824**: p. 39-44.
20. Sher, N., et al., *Simultaneous determination of antihistamine anti-allergic drugs, cetirizine, domperidone, chlorphenamine maleate, loratadine, meclizine and buclizine in pharmaceutical formulations, human serum and pharmacokinetics application.* Analytical Methods, 2014. **6**(8): p. 2704.
21. Kuminek, G., et al., *Development and validation of a stability-indicating HPLC method for the determination of buclizine hydrochloride in tablets and oral suspension and its application to dissolution studies.* Química Nova, 2012. **35**(1).
22. Arayne, M.S., et al., *Simultaneous determination of gliquidone, fexofenadine, buclizine, and levocetirizine in dosage formulation and human serum by RP-HPLC.* Journal of Chromatographic Science, 2010. **48**.
